# Supplementary material for: Electrochemical Response of 3D-Printed Free-Standing Reduced Graphene Oxide Electrode for Sodium Ion Batteries Using a Three-Electrode Glass Cell
Source: Materials (Basel). 2023 Jul 31;16(15):5386. doi: 10.3390/ma16155386 (PMC10419660; doi:10.3390/ma16155386)
Supplement: Supplementary file 1 [file materials-16-05386-s001.zip › materials-2490677-supplementary.pdf]

Supplementary Information

Electrochemical Response of 3D-Printed Free-Standing Reduced Graphene Oxide Electrode for Sodium Ion Batteries Using a Three-Electrode Glass Cell

Cristina Ramírez [1,*], María Isabel Osendi [1], Juan José Moyano [1,2], Jadra Mosa [1] and Mario Aparicio [1]

[1] Institute of Ceramics and Glass (ICV),Consejo Superior de Investigaciones Científicas, CSIC, Kelsen 5, Cantoblanco, 28049 Madrid, Spain
[2] Universidad Politécnica de Madrid, 28040 Madrid, Spain
* Correspondence: cristina.ramirez@icv.csic.es

XRD analysis of 3D rGO electrodes after electrochemical testing

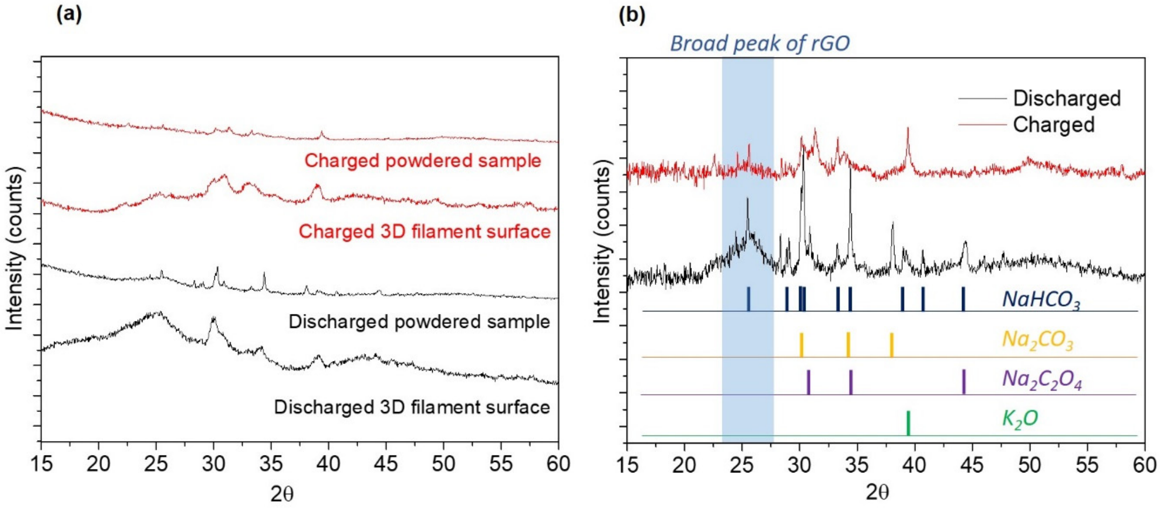

Figure S1. XRD patterns of the charged and discharged 3D rGO electrodes. **a)** Comparison between the patterns obtained from the surface of filaments in the 3D structure´s top layer and from the corresponding crushed samples, using similar acquisition parameters. Filaments show four main broad peaks that are better resolved in the crushed samples. **b)** Identification of main phases in powdered samples. Both discharged and charged rGO electrodes presented varied Na-rich reaction products associated with electrolyte decomposition as nahcolite ($NaHCO_3$), sodium carbonate (Na_2CO_3), or natroxalate ($Na_2C_2O_4$).

Reaction products in 3D rGO charged and discharged samples

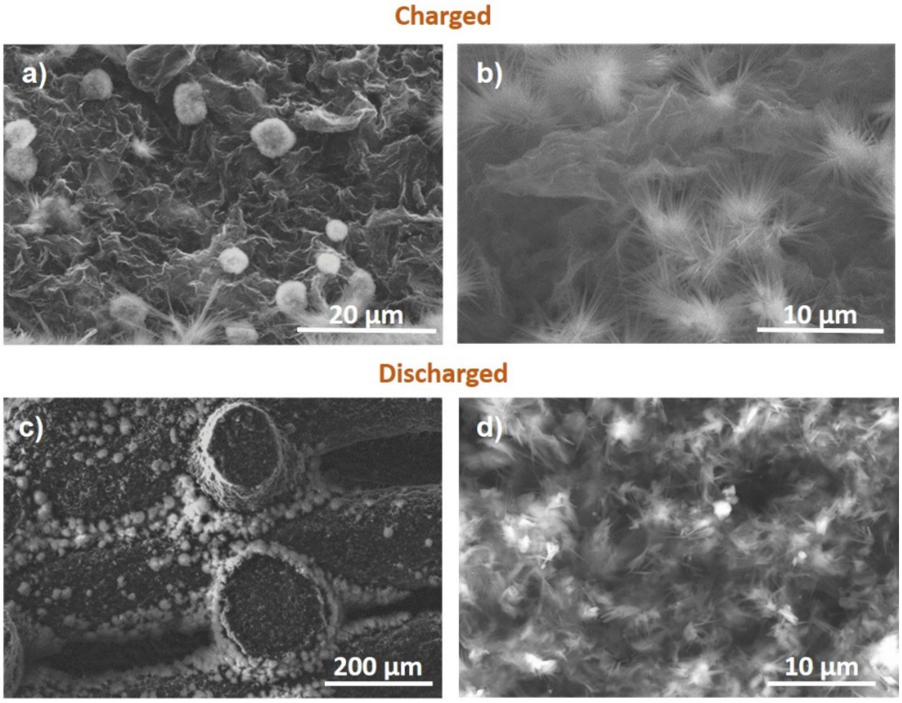

Figure S2. SEM images of the sodium compounds coming from the electrolyte observed at the surface of 3D rGO structures after the electrochemical test. **a), b)** charged sample, **c), d)** discharged sample.

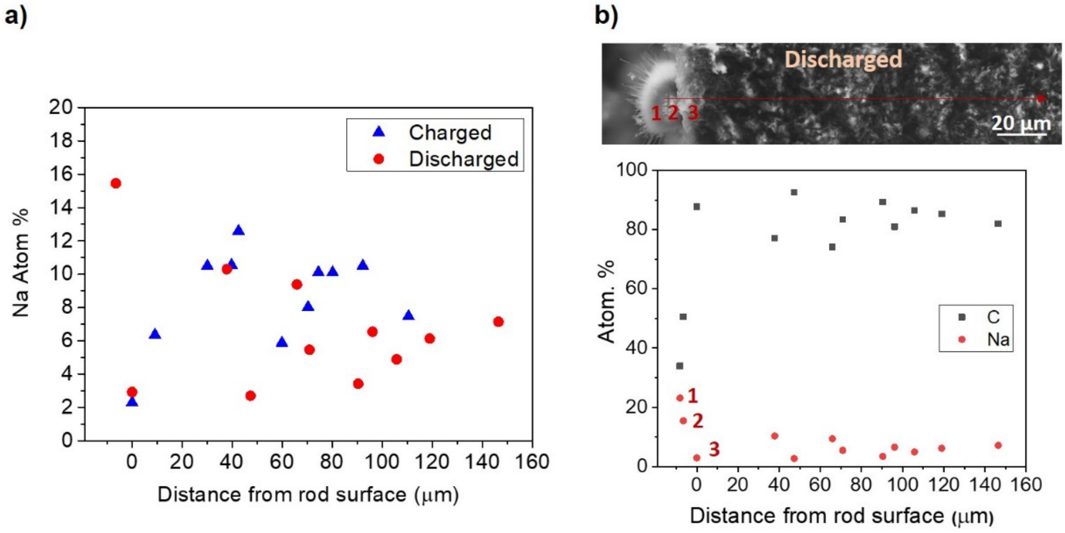

Figure S3. a) EDS microanalysis comparing Na quantification across the filaments for charged and discharged electrodes, and **b)** microanalyses on the cross-section of the 3D rGO discharged sample showing the concentration of Na on the filament surface.

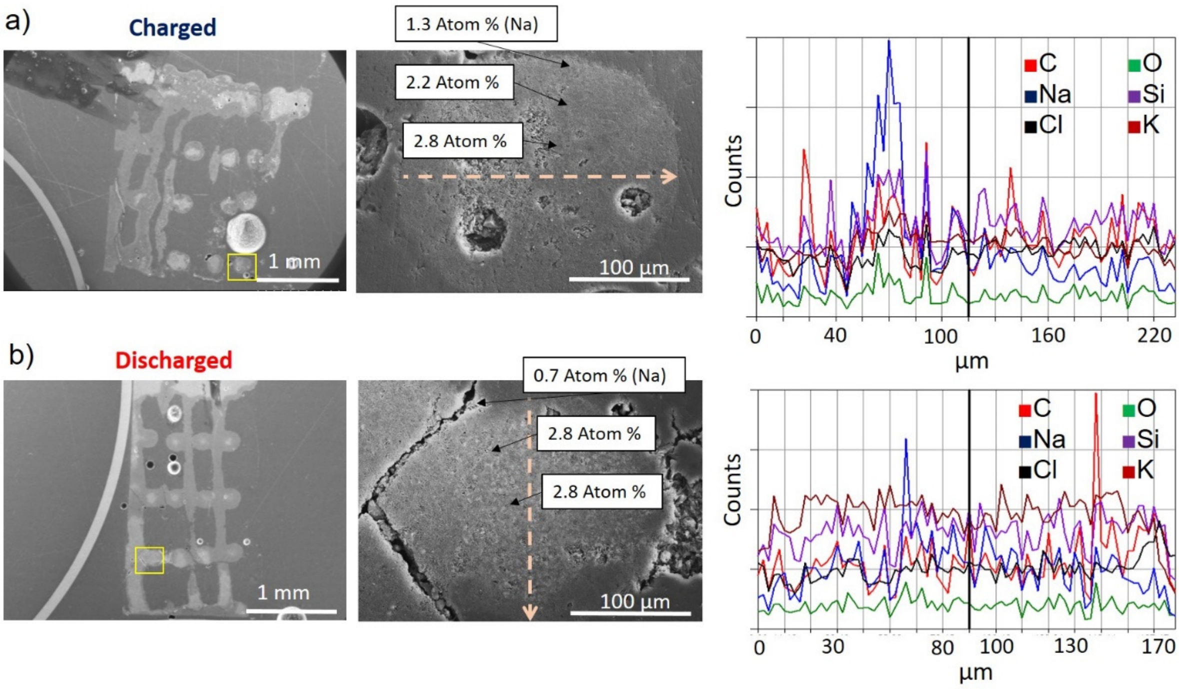

Figure S4. SEM micrograph of polished 3D rGO samples embedded in epoxy resin. **a)** charged 3D rGO at low magnification, high magnification on filament (marked with a yellow square) with Na atom. % at different points, and EDS line-scan across the filament diameter. **b)** Same for the discharged 3D rGO sample.

<u>EDS elemental mapping</u>

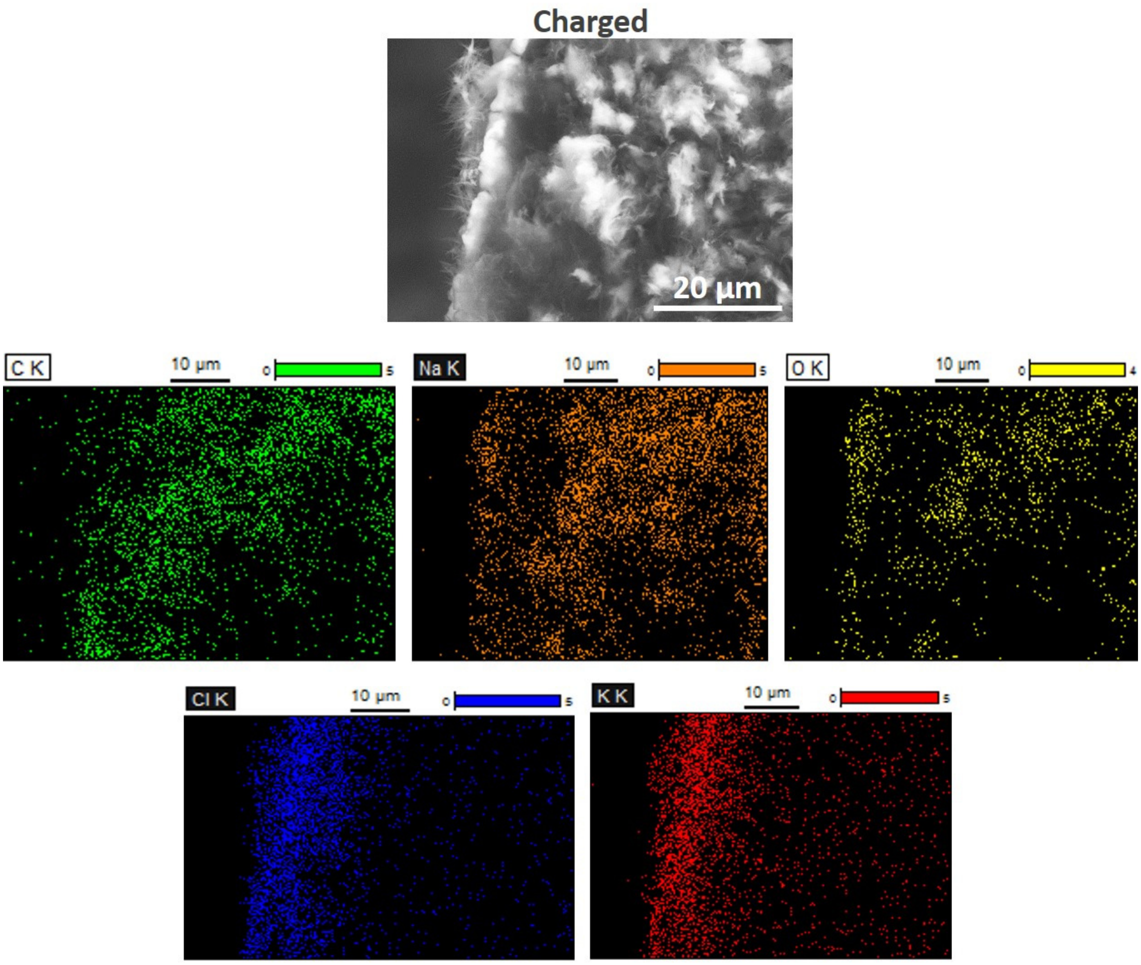

Figure S5. Spectral images for C, Na and O elements of a filament cross-section near the surface for charged rGO electrode. Maps for Cl and K are included as well.

EDS maps of the cross section of filament cross-section in the 3D rGO discharged electrode is represented in Fig. S6 indicating the strong Na and Cl concentration on the surface.

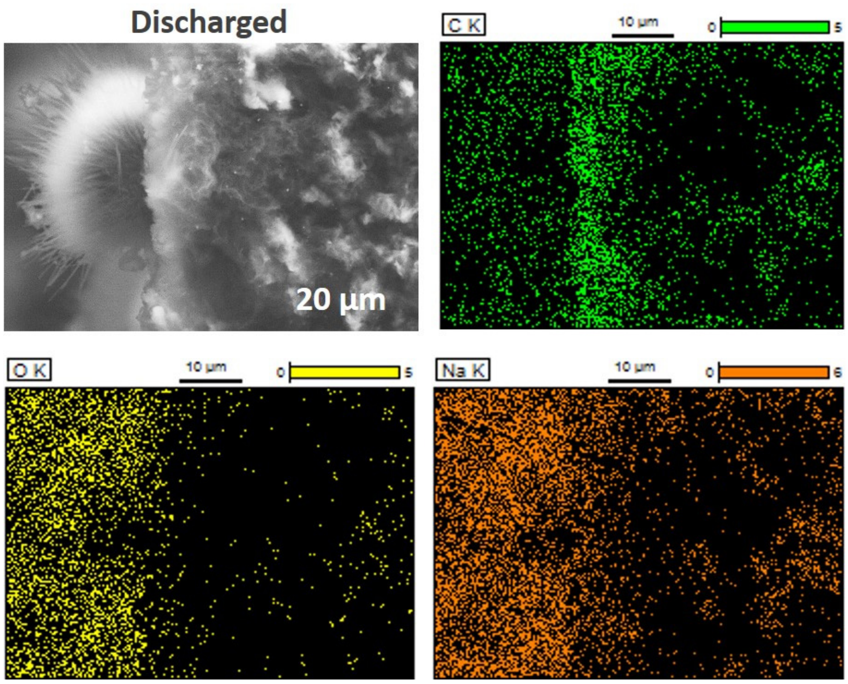

Figure S6. EDS elemental maps of discharged 3D rGO sample.

Spectral EDS maps performed in 3D rGO filaments after 1200 ºC reducing treatment in N_2 (Fig. S7) reveal the massive C content (99.5 wt%), and residual presence of Cl, K, Ca, and Mn, homogenously distributed, that are contaminants attributable most probably to the GO synthesis. The residual presence of oxygen is in agreement with the reported combustion elemental analysis (< 2 wt%).

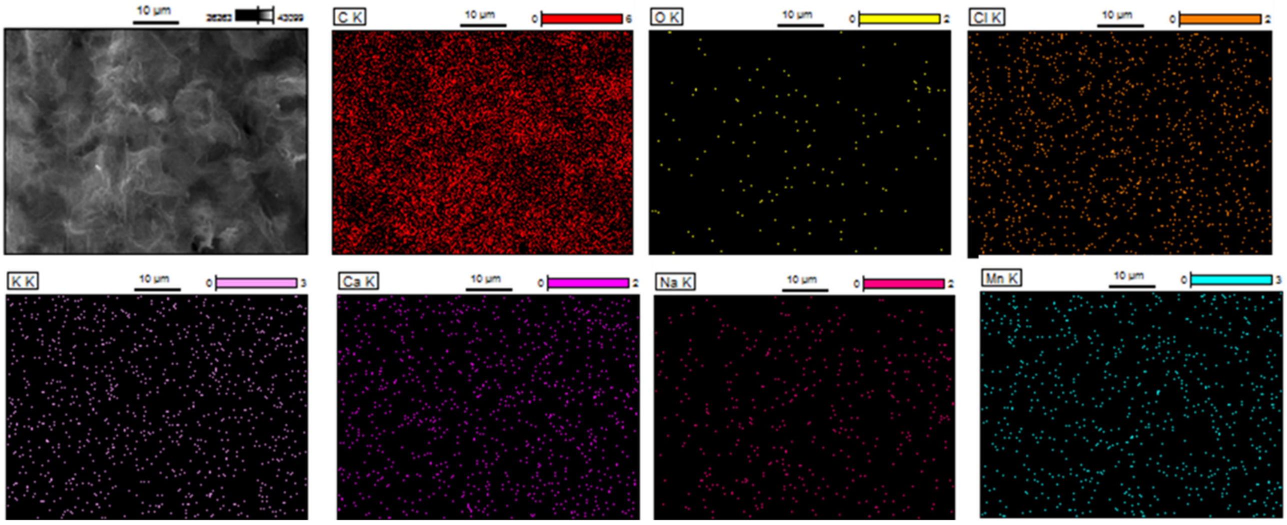

Figure S7. EDS elemental maps from original 3D rGO filament cross-section after reduction treatment at 1200 °C. Carbon distribution is presented in the first map in red color, followed by O, Cl, K, Ca, Na, and Mn.

TEM analysis of charged rGO electrode

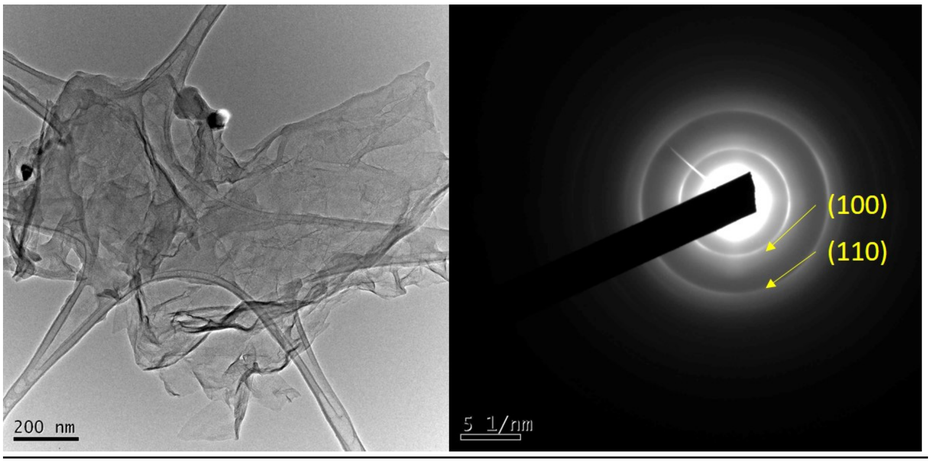

Figure S8. TEM image of rGO sheets and corresponding EDP of the zone. Charged samples do not show a high concentration of particles as in the case of discharged electrode.

Raman spectroscopy of unpolished rGO samples

Raman spectroscopy area scans were taken on the filament surface of the electrodes (unpolished samples). An example is shown in Fig. S9 for discharged 3D rGO sample. The image built partially reproduces the filamentary product frequently observed on the surface of the filaments (Fig. S2c). The single spectrum from the marked position (red) in the optical image displays the typical rGO bands (D, G and 2D) and a low-intensity peak at 1068 cm^{-1}. This peak was not observed in the polished samples because of the utmost surface removal during polishing.

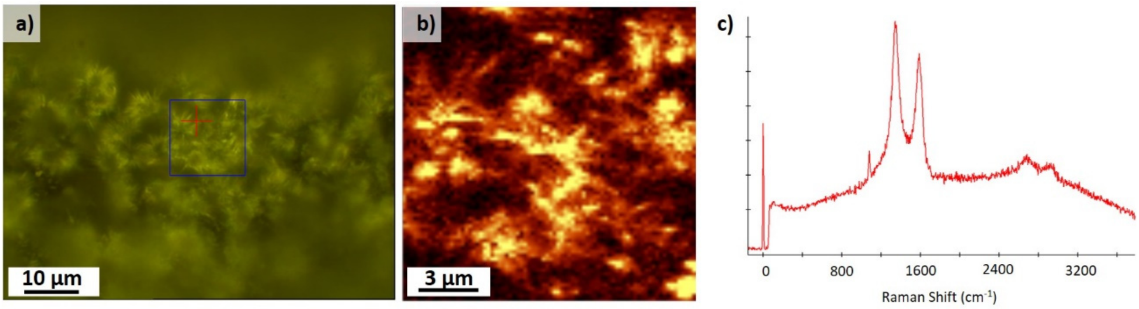

Figure S9. a) Optical image of the filament surface of a 3D rGO discharged electrode marking the scanned region, **b)** intensity map for the peak at 1068 cm^{-1} in the squared area of a). **c)** example of single spectrum from filament surface.

The position of D and G bands of both electrodes averaged on the scanned area is compared with that of the original 3D rGO sample (also surface averaged).

Table S1. Summary of characteristics Raman bands for the electrodes and the original 3D rGO sample.

Sample*	D (cm^{-1})	G (cm^{-1})	D/G
3D rGO Na charged, filament surface	1348 ± 1	1586 ± 4	1.8 ± 0.1
3D rGO Na/ discharged, filament surface	1348 ± 2	1579 ± 5	1.7 ± 0.2
3D rGO original	1354 ± 2	1592 ± 2	1.9 ± 0.2

*unpolished filament surface
